# Pregnancy outcomes following exposure to onabotulinumtoxinA†

**DOI:** 10.1002/pds.3920

**Published:** 2015-12-04

**Authors:** Mitchell F. Brin, Russell S. Kirby, Anne Slavotinek, Mary Ann Miller‐Messana, Lori Parker, Irina Yushmanova, Huiying Yang

**Affiliations:** ^1^ Allergan, Inc.IrvineCAUSA; ^2^ University of South FloridaTampaFLUSA; ^3^ University of CaliforniaSan FranciscoCAUSA; ^4^ University of CaliforniaIrvineCAUSA; ^5^ Now at the Department of Drug Safety and PharmacovigilancePharmacyclics, Inc.SunnyvaleCAUSA

**Keywords:** onabotulinumtoxinA, pregnancy, fetal defects, pharmacoepidemiology

## Abstract

**Purpose:**

To evaluate pregnancy outcomes following onabotulinumtoxinA (US Food and Drug Administration pregnancy category C product) exposure using the Allergan safety database.

**Methods:**

The Allergan Global Safety Database contains reports of onabotulinumtoxinA administration before/during pregnancy, including both prospective (reported before outcome) and retrospective (outcome already known) cases. The database was searched from 1/1/90 to 12/31/13 for eligible cases where treatment occurred during pregnancy or ≤3 months before conception. To minimize reporting bias, prevalence rates were focused on prospective cases.

**Results:**

Of 574 pregnancies with maternal onabotulinumtoxinA exposure, 232 were eligible with known outcomes. Patients received onabotulinumtoxinA most frequently for cosmetic indications (50.5%), movement disorders (16.8%), and pain disorders (14.2%). Of the 137 with dose information, 40.1% received <50U, 14.6% 50U to <100U, 27.7% 100U to <200U, and 17.5% ≥200U. Among 146 cases with known maternal age, 47.9% were ≥35 years. Most (96.0%) fetal exposures occurred during/before the first trimester. Of the 137 prospective cases (139 fetuses), 110 (79.1%) were live births; 29 (20.9%; 95% CI, 14.0–30.0%) ended in fetal loss (21 spontaneous, 8 induced abortions). Among live births, 106 (96.4%) were normal, with four abnormal birth outcomes (1 major fetal defect, 2 minor fetal malformations, 1 birth complication), giving a 2.7% (3/110; 95% CI, 0.6–8.0%) prevalence rate for overall fetal defects.

**Conclusions:**

A 24‐year retrospective review of the Allergan safety database shows that the prevalence of fetal defects in onabotulinumtoxinA‐exposed mothers before/during pregnancy (2.7%) is comparable with background rates in the general population. Pregnancy outcome monitoring in onabotulinumtoxinA‐exposed women continues. © 2015 The Authors. *Pharmacoepidemiology and Drug Safety* published by John Wiley & Sons Ltd.

## Introduction

OnabotulinumtoxinA (BOTOX^®^/BOTOX^®^ Cosmetic, Allergan, Inc., Irvine, CA) has been studied and marketed globally for a variety of indications for over 20 years. In the USA, approved indications for onabotulinumtoxinA include strabismus, blepharospasm, cervical dystonia, axillary hyperhidrosis, chronic migraine, upper limb spasticity, neurogenic detrusor overactivity, overactive bladder, and the cosmetic indications of glabellar lines and lateral canthal lines (Table 1).^1^, ^2^ OnabotulinumtoxinA is indicated for use in adults in the USA, except for the treatment of blepharospasm and strabismus, in which it is indicated for patients ≥12 years of age. Over the past 24 years (1990–2014), approximately 54 million vials of BOTOX^®^ and BOTOX^®^ Cosmetic have been distributed worldwide (Allergan data on file).

**Table 1:** Approved indications for onabotulinumtoxinA in the USA and Europe

Year of FDA approval	Indication	Typical age ranges*	Indication statement approved by FDA^1^, ^2^	Year of EU approval†
1989	Strabismus	Commonly in first decade of life^35^	Treatment of strabismus in patients ≥12 years of age	N/A
1989	Blepharospasm	Onset in 50s,^36^ with prevalence increasing with age^37^	Treatment of blepharospasm associated with dystonia in patients ≥12 years of age	1994
2000	Cervical dystonia	Onset in early 40s^38^, ^39^, ^40^	Treatment of cervical dystonia in adult patients, to reduce the severity of abnormal head position and neck pain	1995
2002‡	Glabellar lines	Mean age of onset, 46.4 ± 9.9 years^41^	Temporary improvement in the appearance of moderate to severe glabellar lines associated with corrugators and/or procerus muscle activity in adult patients	2003
2004	Primary axillary hyperhidrosis	Onset in mid‐20s, with highest prevalence of 3.5–4.5% among those aged 25–64 years^42^	Treatment of severe axillary hyperhidrosis that is inadequately managed by topical agents in adult patients	2001
2010	Focal upper limb spasticity	Prevalence of stroke increases with age: 4.8% among those aged 65–84 years and 7.1% in those >75 years.^43^ At 12 months post‐stroke, prevalence of upper limb spasms is 17–19%	Treatment of upper limb spasticity in adult patients	2001
2010	Chronic migraine	Peak prevalence in 40s at 1.9% for females; 0.8% for males^44^	Prophylaxis of headaches in adult patients with chronic migraine (≥15 days per month with headache lasting 4 h or longer per day)	2010
2011	Detrusor overactivity associated with a neurologic condition	Mean age 62.5 years among NDO patients in a US claims database^45^	Treatment of urinary incontinence because of detrusor overactivity associated with a neurologic condition (e.g., spinal cord injury, multiple sclerosis) in adults who have an inadequate response to or are intolerant of an anticholinergic medication	2011
2013	Overactive bladder	Age group with highest prevalence: ≥60 years (19.1% in females; 8.2% in males)^46^	Treatment of overactive bladder with symptoms of urgency urinary incontinence, urgency, and frequency in adults who have an inadequate response to or are intolerant of an anticholinergic medication	2013
2013‡	Lateral canthal lines	Mean age of onset, 46.4 ± 9.9 years^41^	Temporary improvement in the appearance of moderate to severe lateral canthal lines associated with orbicularis oculi activity in adult patients	2014
N/A	Juvenile cerebral palsy dynamic equinus foot deformity	Appears in infancy or childhood	N/A	1997
N/A	Lower limb spasticity	Prevalence of stroke increases with age: 4.8% among those aged 65–84 years and 7.1% in those >75 years.^43^ At 12 months post‐stroke, prevalence of lower limb spasms is 11–32%	N/A	2014

EU = European Union, FDA = Food and Drug Administration, N/A = not applicable, NDO = neurogenic detrusor overactivity.

Table is adapted from Brin MF, Blitzer A. History of onabotulinumtoxinA therapeutic. In *Botulinum Toxin*. Carruthers J and Carruthers A (eds). 3rd edition. London: Elsevier Saunders, 2013:6–12.

* The mean age of onset for the cosmetic indications is based on treatment data.^41^ For all other indications, the data are as published in the literature, and not necessarily representative of the population being treated.

† First EU approval in UK or Ireland /France. (Reference Member State of the Mutual Recognition Process for Therapeutic/Aesthetic respectively). Specific indications often vary from those licensed in the USA.

‡ As BOTOX^®^ Cosmetic.

OnabotulinumtoxinA is designated as a US Food and Drug Administration (FDA) pregnancy category C pharmaceutical product, as there are no adequate and well controlled studies in pregnant women, and it should only be used during pregnancy if the benefits outweigh the potential risks.^3^ While women who are pregnant, nursing, or planning a pregnancy are excluded from clinical trials on botulinum toxin (BoNT), many women being treated with onabotulinumtoxinA (eg, chronic migraine, axillary hyperhidrosis, and cosmetic use) are of reproductive age. Several of the indications for which onabotulinumtoxinA is approved are more prevalent in women and overall use is greater in females. For example, the prevalence of chronic migraine is 2.5 to 6.5 times higher in women than in men,^4^ >60% of hyperhidrosis patients are reported to be female,^5^ and in the USA, almost 90% of the onabotulinumtoxinA injections for cosmetic use are for women.^6^ In addition, pregnancy rates for women in their 30s and early 40s have increased in the developed countries.^7^

Given the high frequency of onabotulinumtoxinA use among women, especially those of fertile age, we have analyzed birth outcomes of women exposed to onabotulinumtoxinA prior to or during pregnancy.

## Methods

The Allergan Global Safety Database was used to identify pregnancy cases, in which the patient was exposed to onabotulinumtoxinA or BoNT type A (manufacturer unspecified). This database contains individual case safety reports received from pre‐ and post‐approval sources (approved indications shown in Table 1), including Allergan‐sponsored and partner‐sponsored clinical trials (serious adverse events and pregnancies), spontaneous sources (serious and non‐serious events reported by consumers and healthcare providers), post‐authorization studies, health authorities, and published literature. Women enrolled in Allergan‐sponsored clinical trials who had a positive pregnancy test during the trial period were withdrawn from the studies and followed for pregnancy outcome. The database includes cases with pregnancy outcomes that are prospectively (pregnancy case reported before outcome was known) or retrospectively (outcome known when pregnancy case was reported) collected. When a pregnancy exposure is reported to Allergan, a minimum of three attempts are made, by phone, email, and/or letter to gather information on the pregnancy exposure and to obtain follow‐up information on the pregnancy outcome. Additional follow‐up is based upon the reporter's willingness to provide more information, receipt of consent to access medical information, and provision of the doctor's contact information. Whenever possible, the patient's physician is contacted for additional information about the pregnancy.

For this analysis, the database was queried from January 1, 1990 through December 31, 2013 for case reports involving an identifiable patient exposed to onabotulinumtoxinA or BoNT type A who was confirmed pregnant, and the patient age was unknown or was under the age of 65 years. Additionally, case reports concerning accidental topical exposure to onabotulinumtoxinA were excluded.

Only cases in which onabotulinumtoxinA injection occurred during pregnancy or up to 3 months prior to the estimated date of conception were retained; these are considered eligible pregnancies for the purpose of this analysis. OnabotulinumtoxinA is not expected to be present in peripheral blood at measurable levels following intramuscular or intradermal injection at the recommended doses, and preclinical studies^8^, ^9^ predict that any potentially systemic onabotulinumtoxinA would be completely eliminated from the systemic circulation within 48 h post‐injection in rodents. Therefore, it was decided to review cases with maternal exposure of onabotulinumtoxinA 3 months prior to conception, because this is the approximate duration of action of the drug in striated muscle.^10^

All cases were reviewed by a medical safety physician, who determined if the outcome was a normal live birth or fetal loss (which included both spontaneous and elective abortions, as well as other cases of fetal loss), or in the case of a live birth, whether there were any fetal defects. Elective abortion cases included the birth type of “elective termination” or Medical Dictionary for Regulatory Activities preferred term of “abortion induced” or “abortion.” Fetal defects were assigned to the following categories^11^: major, which have serious medical and/or social implications and often require surgical repair; minor, which are of mostly cosmetic significance and are rarely medically significant or require surgical repair; birth complication, which results from the birthing process; genetic abnormality, which results from a genetic disorder; and significant adverse event, which is another event considered to be significant by the reviewer. Outcomes of interest were fetal defects and fetal loss. Additional evaluated variables were maternal age, timing of exposure, indication, and dose.

Retrospective pregnancy cases (ie, reported after the pregnancy outcome is known) have an inherent reporting bias because they may be influenced by the outcome itself; for example, abnormal outcomes are more likely to be reported than normal outcomes. Such bias may lead to a higher proportion of abnormal outcomes that does not reflect the “true” prevalence rate. As noted by the FDA^12^ and other published reports,^13^, ^14^, ^15^ retrospective reports will not provide an accurate risk calculation. For completeness, however, the proportions of abnormal pregnancy outcomes among retrospective cases are provided. In cases of prospective pregnancies, reporting bias regarding outcome is not anticipated because the outcome has not yet occurred. Consistent with FDA guidance^12^ and other reports^13^, ^14^, ^15^ only prospective pregnancy cases are used to estimate prevalence rates of abnormal pregnancy outcomes, and their 95% confidence intervals (CI), based on a Poisson distribution, are provided.

## Results

### Distribution of cases

**Figure 1:**
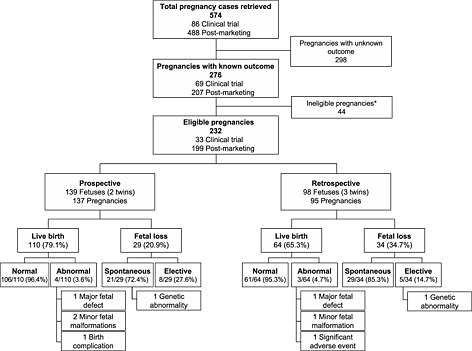
Distribution of pregnancy cases. *Pregnancies in which onabotulinumtoxinA injection occurred >3 months before the estimated date of conception

Overall, 574 pregnancy cases were retrieved from the Allergan Global Safety Database for the time period of January 1, 1990 through December 31, 2013 (Figure 1). Out of these, 276 pregnancies (48.1%) had a known outcome, and of these 276 cases, 232 pregnancies (137 prospective and 95 retrospective) were eligible and encompassed 237 fetal records (139 prospective and 98 retrospective). Six of the overall 574 pregnancy cases (two prospective and four retrospective) were exposed to unspecified BoNT.

### Maternal characteristics

Maternal ages were known for 146 (62.9%) of the total known outcome cases (Table 2). Of the patients with known age, 47.9% were of advanced maternal age (≥35 years); 42.3% of prospective patients were ≥35 years and 59.2% of retrospective patients were ≥35 years. The most commonly reported condition for which patients received onabotulinumtoxinA injection was cosmetic use (50.5%), followed by movement disorders (16.8%), pain disorders (14.2%), and hyperhidrosis (11.6%) (Table 2). The majority (82.6%) of fetal exposures occurred during the first trimester (Table 2). Of the total patients with a known dose (total dose), 40.1% received <50U, 14.6% received 50U to <100U, and 45.3% received ≥100U (Table 2).

**Table 2:** Characteristics of prospective and retrospective pregnancy cases with known outcome*

	Prospective pregnancies (*n* = 137)	Retrospective pregnancies (*n* = 95)	Total (*N* = 232)
Maternal age			
Age known, *n*	97	49	146
<35 years	56 (57.7)	20 (40.8)	76 (52.1)
≥35 years	41 (42.3)	29 (59.2)	70 (47.9)
BOTOX indication *MedDRA preferred term*			
Indication known, *n*	128	62	190
Cosmetic	70 (54.7)	26 (41.9)	96 (50.5)
*Skin cosmetic procedure*	1	0	1
*Skin wrinkling*	69	26	95
Gastrointestinal disorders	1 (0.8)	2 (3.2)	3 (1.6)
*Anal fissure*	1	0	1
*Esophageal achalasia*	0	2	2
Hyperhidrosis	12 (9.4)	10 (16.1)	22 (11.6)
*Hyperhidrosis*	12 (9.4)	10 (16.1)	22 (11.6)
Movement disorders	15 (11.7)	17 (27.4)	32 (16.8)
*Blepharospasm*	1	0	1
*Dystonia*	4	0	4
*Hemiparesis*	1	0	1
*Muscle contracture*	0	1	1
*Spasmodic dysphonia*	1	2	3
*Strabismus*	0	1	1
*Torticollis*	8	13	21
Pain disorders	22 (17.2)	5 (8.1)	27 (14.2)
*Headache*	2	0	2
*Migraine*	17	5	22
*Muscle spasms*	2	0	2
*Pelvic pain*	1	0	1
Spasticity	3 (2.3)	1 (1.6)	4 (2.1)
*Muscle spasticity*	3	1	4
Urological disorders	5 (3.9)	1 (1.6)	6 (3.2)
*Cystitis interstitial*	1	0	1
*Hypertonic bladder*	1	0	1
*Neurogenic bladder*	3	1	4
Timing of exposure			
Timing known, *n*	124	77	201
Prior to conception			
>2–3 months	2 (1.6)	1 (1.3)	3 (1.5)
>1–2 months	7 (5.6)	2 (2.6)	9 (4.5)
0–1 month	12 (9.7)	3 (3.9)	15 (7.5)
First trimester	98 (79.0)	68 (88.3)	166 (82.6)
Second trimester	5 (4.0)	1 (1.3)	6 (3.0)
Third trimester	0 (0)	2 (2.6)	2 (1.0)
BOTOX dose			
Dose known, n	95	42	137
<50U	40 (42.1)	15 (35.7)	55 (40.1)
50U to <100U	13 (13.7)	7 (16.7)	20 (14.6)
100U to <150U	17 (17.9)	9 (21.4)	26 (19.0)
150U to <200U	10 (10.5)	2 (4.8)	12 (8.8)
200U to <250U	6 (6.3)	3 (7.1)	9 (6.6)
250U to <300U	1 (1.1)	0 (0)	1 (0.7)
300U to <350U	6 (6.3)	1 (2.4)	7 (5.1)
350U to <400U	2 (2.1)	0 (0)	2 (1.5)
>400U	0 (0)	5 (11.9)	5 (3.6)

MedDRA = Medical Dictionary for Regulatory Activities.

* Data are expressed as *n* or *n* (% among those with known information).

### Fetal outcomes

#### Overview

There were 137 prospective pregnancies, comprising 139 fetuses because of two sets of twins (Figure 1). Of these, 110 (79.1%) resulted in a live birth. Most (96.4%) of the live births were normal. There were four abnormal birth outcomes, including one major fetal defect, two minor fetal malformations, and one birth complication (Table 3). The prevalence rate for major fetal defects was 0.9% (1/110; 95% CI, 0.02–5.1%) and for overall fetal defects it was 2.7% (3/110; 95% CI, 0.6–8.0%). Of the prospective fetuses, 20.9% (29/139; 95% CI, 14.0–30.0%) ended in fetal loss. Twenty‐one fetal losses (72.4%) were spontaneous, one of which included a genetic abnormality, and eight (27.6%) were elective abortions.

**Table 3:** Summary of fetal defects in prospective and retrospective cases of live births and abortions

Adverse event	Outcome	BOTOX indication	Time of exposure	BOTOX dose	Maternal age
Prospective fetal defects in live births					
Major fetal defects (*n* = 1)					
Ventricular septal defect	C‐section; asymptomatic, no intervention required	Axillary hyperhidrosis	37 days pre‐conception	100U	28 years
Minor fetal malformations (*n* = 2)					
Metatarsus adductus	Induced vaginal delivery (decreased fetal movement); no other abnormalities	Chronic migraine	15 days post‐conception	155U	19 years
Innocent asymptomatic cardiac murmur*	Family history of cardiac murmur	Blepharospasm	Trimesters 1, 2, 3	8U, 12U, 14U	23 years
Birth complication (*n* = 1)					
Horner syndrome	C‐section, placenta previa, and uterine varicosities; no abnormalities reported 11 months later	Axillary hyperhidrosis	“Few days” before conception	100U (50U each axilla)	Unknown
Prospective fetal defect in abortions					
Genetic abnormalities (*n* = 1)					
Down syndrome	Miscarriage at gestation month 5	Skin wrinkling (glabella)	40 days post‐conception	12U	38 years
Retrospective fetal defects in live births					
Major fetal defects (*n* = 1)					
Tracheoesophageal fistula, esophagealatresia	Pre‐term labor at ~33 weeks resulting in C‐section; surgical repair successful	Facial wrinkles	3 days post‐conception	36U	30 years
Minor fetal malformations (*n* = 1)					
Laryngomalacia	Planned C‐section at 38 weeks; settled spontaneously over several months	Facial wrinkles	1 week pre‐conception and 2 weeks post‐conception	Not reported	38 years
Significant adverse event (*n* = 1)					
Brain tumor (unspecified)†	Successful surgical resection	Cervical dystonia	Within 1 week before conception	100U	25 years
Retrospective fetal defects in abortions					
Genetic abnormality (*n* = 1)					
Down syndrome	Elective abortion at week 20	Skin wrinkling (glabella)	“Immediately after injection”	25U	40 years

* Diagnosed at 7 days.

† Diagnosed at 13 months.

There were 95 retrospective pregnancies and 98 fetuses because of three sets of twins (Figure 1), 64 (65.3%) of which resulted in a live birth. Out of the 64 live births, 61 (95.3%) were normal. The three abnormal birth outcomes included one major fetal defect, one minor fetal malformation, and one significant adverse event (Table 3). Of the 98 fetuses from retrospective pregnancies, 34 (34.7%) ended in fetal loss, 29 (85.3%) were spontaneous losses, and 5 (14.7%) were elective abortions, one of which included a genetic abnormality.

#### Fetal loss

Overall, there were 50 spontaneous abortions in the 47 prospective and retrospective pregnancies (Table S1). The most commonly reported condition for which patients experienced spontaneous fetal loss was cosmetic (64.4%), followed by movement disorders (13.3%), pain disorders (13.3%), and hyperhidrosis (6.7%). The most common dose of onabotulinumtoxinA among patients who experienced spontaneous fetal loss was <50U, and the majority (94.3%) of fetal exposure occurred during the first trimester. The most common gestational ages at the time of spontaneous loss were between 1 and 2 months (43.2%) and 2 and 3 months (43.2%). Most (26 [70.3%]) of the spontaneous abortions had the documented risk factor of a maternal age >35 years.^16^ One spontaneous abortion involved a genetic abnormality (Down syndrome); more information on the case is provided in Table 3.

Among the prospective pregnancy cases, a significantly higher proportion of patients with spontaneous abortions (68.8%) were of advanced maternal age (ie, ≥35 years) compared with those experiencing live births (39.5%; *p* = .032) (Table 4). A majority of the patients with spontaneous abortions (89.5%) reported receiving onabotulinumtoxinA for cosmetic use, which was comparable to the overall rate of cosmetic (vs. therapeutic) use of 85.8% on a per‐patient basis in the US population (data on file). Cosmetic use of onabotulinumtoxinA was reported in 50.5% of the patients with live births. It is notable, however, that the timing of exposure to onabotulinumtoxinA was comparable in both the spontaneous abortions and live birth cases (Table 4).

**Table 4:** Comparison of characteristics between prospective pregnancy cases with live births and spontaneous abortions*

	Pregnancies with live births (*n* = 109)	Pregnancies with spontaneous abortions (*n* = 20)	*p*‐values
Maternal age			
Age known, *n*	76	16	0.032
<35 years	46 (60.5)	5 (31.3)	
≥35 years	30 (39.5)	11 (68.8)	
BOTOX indication			
Indication known, *n*	103	19	0.002
Cosmetic	52 (50.5)	17 (89.5)	
Therapeutic	51 (49.5)	2 (10.5)	
Timing of exposure			
Timing known, *n*	99	17	0.518
Prior to conception	18 (18.2)	2 (11.8)	
First/Second trimester	81 (81.8)	15 (88.2)	
BOTOX dose			
Dose known, *n*	80	12	0.141
<50U	32 (40.0)	8 (66.7)	
50U to <100U	11 (13.8)	2 (16.7)	
≥100U	37 (46.3)	2 (16.7)	

Pregnancy cases with unknown maternal age, BOTOX indication, timing of exposure or BOTOX dose were not included in the individual analyses.

* Data are expressed as *n* or *n* (% among those with known information).

There were a total of 13 elective abortions from both prospective and retrospective cases, which occurred with similar frequency among patients treated for various indications (Table S2). The majority (81.8%) of fetal exposures in these cases occurred during the first trimester. Of the 10 cases for which the time of abortion was known, nine occurred in the first trimester. The reasons for elective abortions were known in the following nine cases: five personal/social, one fetal disorder, one high risk because of age, one blighted ovum, and one gestational sac with no embryo. One elective abortion involved the genetic abnormality Down syndrome (Table 3).

#### Fetal defects

Among all live births (*n* = 174), there were a total of seven abnormalities (Table 3). Two fetal cases were categorized as a major malformation: ventricular septal defect, which was prospective, asymptomatic, and did not require medical intervention, and tracheoesophageal fistula/esophageal atresia, a retrospective case that was successfully surgically repaired. The single prospective case of major malformation gave a 0.9% (1/110; 95% CI, 0.02–5.1%) prevalence rate of major fetal defects.

There were three minor fetal malformations: laryngomalacia, which resolved spontaneously over several months, and a reportedly asymptomatic innocent cardiac murmur in an infant with a family history of cardiac murmur. One prospective case with metatarsus adductus was provisionally classified as having a minor fetal defect, although data regarding the severity and need for interventions were not known for this case.

The one birth complication was Horner syndrome, which spontaneously resolved after 11 months. The one significant adverse event was a benign brain tumor identified at 13 months, which was successfully surgically resected.

## Discussion

This analysis examined over 20 years of data reported to Allergan on pregnancy outcomes in women exposed to onabotulinumtoxinA or unspecified BoNT. We observed that the rates of spontaneous abortion and fetal defects among pregnant women treated with onabotulinumtoxinA are similar to those reported for the general population. Among prospective cases, the observed prevalence rates of elective and spontaneous abortion were 5.8% (8/139; 95% CI, 2.5–11.3%) and 15.1% (21/139; 95% CI, 9.4–23.1%), respectively, compared with rates of 18.4 and 17.0% for elective and spontaneous abortions in the US general population.^7^ As expected, the retrospectively collected prevalence rates of elective and spontaneous abortion outcomes were higher, owing to the inherent reporting bias seen in a retrospective subset. As noted by the FDA^12^ and other authors,^13^, ^14^, ^15^ the retrospective data cannot be compared with prevalence rates in the general population.

In this analysis, the observed prevalence rate of any fetal defects in prospective cases is 2.7% (95% CI, 0.6–8.0%), and the prevalence rate of major fetal defects is 1.8%, which is comparable to large population‐based studies. For example, the Centers for Disease Control and Prevention found a 3% overall prevalence rate of live births with major fetal defects from 1978 to 2005,^17^ and a UK study reported a 2% prevalence rate of major fetal defects diagnosed before 1 year of age in children born between 1990–2009.^18^ The March of Dimes estimates a birth prevalence rate of approximately 6% of the annual births worldwide for serious fetal defects of genetic or partially genetic origin; about 5.5% in high‐income countries.^19^

In addition, these data are consistent with observations from preclinical registration studies conducted by Allergan, in which onabotulinumtoxinA was not selectively toxic to fetal development, and fetal effects were observed only in association with maternal toxicity. No adverse effects on fetal development were observed when pregnant rats received single intramuscular injections of onabotulinumtoxinA 1, 4, or 16U/kg prior to implantation, at implantation, or during organogenesis.^1^ Intramuscular daily injections of onabotulinumtoxinA to pregnant rats or rabbits during the period of organogenesis (total of 12 doses in rats, 13 doses in rabbits) resulted in reduced fetal body weights and decreased fetal skeletal ossification at the two highest doses in rats (4U/kg, 8U/kg) and at the highest dose in rabbits (0.5 U/kg).^1^ These doses were also associated with maternal toxicity, including abortions, early deliveries, and maternal death. The developmental no‐effect doses in these studies were lower than the average high dose used in humans. In addition to the studies on rats and rabbits, onabotulinumtoxinA did not appear to have an effect on neural tube development in a chick embryo model.^20^

Reports in the literature of exposure to BoNT from botulism show low levels of adverse effects on the fetus (Table S3). Clinical botulism did not appear to directly cause any adverse effects on the pregnancy or fetus in six published case reports.^21^, ^22^, ^23^, ^24^, ^25^, ^26^ In one case, the patient was paralyzed and the fetus was the only visible motion, indicating that peripherally circulating BoNT does not cross the placenta.^22^ Indeed, this conclusion was confirmed by an *in vivo* rabbit study,^27^ and is consistent with the notion that should any onabotulinumtoxinA have been present in the systemic circulation after treatment, the large molecular weight makes it unlikely to passively diffuse across the placenta.

It is believed that little systemic distribution of therapeutic doses of onabotulinumtoxinA occurs. OnabotulinumtoxinA is not expected to be present in the peripheral blood at measurable levels following intramuscular or intradermal injection at the recommended doses. The recommended quantities of neurotoxin administered at each treatment session are not expected to result in systemic, overt, distant clinical effects (ie, muscle weakness) in patients without other neuromuscular dysfunction.

Literature reports of BoNT exposure from cosmetic or therapeutic use also show low levels of adverse effects on the fetus. Of the 30 cases of BoNT exposure for cosmetic or therapeutic purposes during pregnancy reported in the literature, two resulted in miscarriages (one of which is included in this analysis^28^) and one in elective abortion with no report of fetal defects.^26^, ^28^, ^29^, ^30^, ^31^, ^32^, ^33^, ^34^

This study has several limitations, which are consistent with other safety database analyses. It is restricted to cases reported to Allergan, and case reports may not include full data on factors such as dose and timing of exposure, which limits detailed analysis on risk factors in this population. In addition, outcomes at the time of birth were examined, but there could also be more subtle developmental effects identified at later postnatal visits.

## Conclusions

Based on a 24‐year retrospective review of the Allergan safety database, the prevalence of abnormal pregnancy outcomes in mothers exposed to onabotulinumtoxinA prior to or during pregnancy is consistent with background rates in the general population. No consistent types of malformation by organ were observed, and no new safety concerns were identified. Allergan will continue to monitor the pregnancy outcomes in onabotulinumtoxinA‐exposed women through routine pharmacovigilance.

## Conflict of Interest

MFB, MMM, LP, and IY are employees of Allergan, Inc. HY was an employee of Allergan, Inc. at the time the analysis was conducted. RSK and AS have previously served as consultants for Allergan, Inc.

**Key Points**

- Several indications for which onabotulinumtoxinA is approved are more prevalent in women, and many women being treated are of reproductive age
- This analysis of reported pregnancies during 24 years of onabotulinumtoxinA utilization shows that the prevalence of abnormal pregnancy outcomes in women exposed to onabotulinumtoxinA is comparable with background rates in the general population
- No consistent types of malformation by organ were observed, and no new safety concerns were identified
- Monitoring of pregnancy outcomes in onabotulinumtoxinA‐exposed women is ongoing

## Supporting information

Supporting info item
