# Supplementary material for: Pregnancy outcomes following exposure to onabotulinumtoxinA
Source: Pharmacoepidemiol Drug Saf. 2015 Dec 4;25(2):179–87. doi: 10.1002/pds.3920 (PMC5063122; doi:10.1002/pds.3920)
Supplement: Supplementary file 1 — Supporting info item [file PDS-25-179-s001.docx]

Supplemental Table 1. Characteristics of women who experienced spontaneous abortions*

|  | Prospective pregnancies  (*n* = 20) | Retrospective pregnancies  (*n* = 27) | Total  (*N* = 47) |
| --- | --- | --- | --- |
| Maternal age |  |  |  |
| Age known, *n* | 16 | 21 | 37 |
| <35 years | 5 (31.3) | 6 (28.6) | 11 (29.7) |
| ≥35 years | 11 (68.8) | 15 (71.4) | 26 (70.3) |
| **BOTOX indication**  *MedDRA preferred term* |  |  |  |
| Indication known, *n* | 19 | 26 | 45 |
| **Cosmetic** | 17 (89.5) | 12 (46.2) | 29 (64.4) |
| *Skin wrinkling* | 17 | 12 | 29 |
| **Hyperhidrosis** | 0 (0) | 3 (11.5) | 3 (6.7) |
| *Hyperhidrosis* | 0 (0) | 3 (11.5) | 3 (6.7) |
| **Movement disorders** | 0 (0) | 6 (23.1) | 6 (13.3) |
| *Spasmodic dysphonia* | 0 | 2 | 2 |
| *Torticollis* | 0 | 4 | 4 |
| **Pain disorders** | 2 (10.5) | 4 (15.4) | 6 (13.3) |
| *Migraine* | 2 | 4 | 6 |
| **Spasticity** | 0 (0) | 1 (3.8) | 1 (2.2) |
| *Muscle spasticity* | 0 | 1 | 1 |
| Timing of exposure |  |  |  |
| Timing known, *n* | 17 | 18 | 35 |
| Prior to conception | 2 (11.8) | 0 (0) | 2 (5.7) |
| First trimester | 15 (88.2) | 18 (100.0) | 33 (94.3) |
| BOTOX dose |  |  |  |
| Dose known, *n* | 12 | 16 | 28 |
| <50U | 8 (66.7) | 9 (56.3) | 17 (60.7) |
| 50U to <100U | 2 (16.7) | 1 (6.3) | 3 (10.7) |
| >100U | 2 (16.7) | 6 (37.5) | 8 (28.6) |
| Gestational age at time of abortion | |  |  |
| Age known | 16 | 21 | 37 |
| First trimester |  |  |  |
| <1 month | 1 (6.3) | 0 (0) | 1 (2.7) |
| 1 to <2 months | 4 (25.0) | 12 (57.1) | 16 (43.2) |
| 2 to <3 months | 9 (56.3) | 7 (33.3) | 16 (43.2) |
| Second trimester | 2 (12.5) | 2 (9.5) | 4 (10.8) |
| Third trimester | 0 (0) | 0 (0) | 0 (0) |

MedDRA = Medical Dictionary for Regulatory Activities.

*Data are expressed as *n* or *n* (% among those with known information).

Supplemental Table 2. Characteristics of women who experienced elective abortions*

|  | Prospective pregnancies  (*n* = 8) | Retrospective pregnancies  (*n* = 5) | Total  (*N* = 13) |
| --- | --- | --- | --- |
| Maternal age |  |  |  |
| Age known, *n* | 5 | 4 | 9 |
| <35 years | 5 (100.0) | 1 (25.0) | 6 (66.7) |
| ≥35 years | 0 (0) | 3 (75.0) | 3 (33.3) |
| **BOTOX indication**  *MedDRA preferred term* |  |  |  |
| Indication known, n | 6 | 4 | 10 |
| **Cosmetic** | 1 (16.7) | 2 (50.0) | 3 (30.0) |
| *Skin wrinkling* | 1 | 2 | 3 |
| **Hyperhidrosis** | 1 (16.7) | 0 (0) | 1 (10.0) |
| *Hyperhidrosis* | 1 (16.7) | 0 (0) | 1 (10.0) |
| **Pain disorders** | 0 (0) | 1 (25.0) | 1 (10.0) |
| *Migraine* | 0 | 1 | 1 |
| **Spasticity** | 2 (33.3) | 0 (0) | 2 (20.0) |
| *Muscle spasticity* | 2 | 0 | 2 |
| **Urological disorders** | 2 (33.3) | 1 (25.0) | 3 (30.0) |
| *Neurogenic bladder* | 2 | 1 | 3 |
| Timing of exposure |  |  |  |
| Timing known, *n* | 8 | 3 | 11 |
| Prior to conception | 1 (12.5) | 1 (33.3) | 2 (18.2) |
| First trimester | 7 (87.5) | 2 (66.7) | 9 (81.8) |
| BOTOX dose |  |  |  |
| Dose known, *n* | 3 | 3 | 6 |
| <50U | 0 (0) | 2 (66.7) | 2 (33.3) |
| >100U | 3 (100.0) | 1 (33.3) | 4 (66.7) |
| Gestational age at time of abortion | |  |  |
| Age known, *n* | 6 | 4 | 10 |
| First trimester | 6 (100.0) | 3 (75.0) | 9 (90.0) |
| Second trimester | 0 (0) | 1 (25.0) | 1 (10.0) |
| Reason for abortion |  |  |  |
| Reason known, *n* | 5 | 4 | 9 |
| Personal/social | 4 (80.0) | 1 (25.0) | 5 (55.6) |
| Fetal disorder | 0 (0) | 1 (25.0) | 1 (11.1) |
| High risk due to age | 1 (20.0) | 0 (0) | 1 (11.1) |
| Blighted ovum | 0 (0) | 1 (25.0) | 1 (11.1) |
| Gestational sac with no embryo | 0 (0) | 1 (25.0) | 1 (11.1) |

MedDRA = Medical Dictionary for Regulatory Activities.

*Data are expressed as *n* or *n* (% among those with known information).

Supplemental Table 3. Summary of pregnancy and fetal complications in cases of clinical botulism

| Publication | Route/Serotype | Maternal age/gestation | Botulism/Severity | Pregnancy complications | Fetal complications/anomalies |
| --- | --- | --- | --- | --- | --- |
| St Clair (1975)^21^ | “Outbreak”  Serum: type A | 32 years/34 weeks | Required vent assist; treated with antitoxin | Partial placental abruption suspected; precipitous delivery at 34 weeks | Intraventricular hemorrhage, hydrocephalus, developmental delay, blindness |
| Polo (1996)^22^ | Green beans  type u/k | 37 years/23 weeks | Tetraparesis; required vent assist; treated with antitoxin | Spontaneous delivery at 33 weeks | None |
| Robin (1996)^23^ | Stinkfish: type A  Stool: type A and E | 24 years/16 weeks | Forced vital capacity at 76%; treated with antitoxin | Spontaneous delivery at 42 weeks | None |
| Morrison (2006)^24^ | IV Drug Abuse  Serum type A | 23 years/36 weeks; history of heroin use | Deteriorating ventilatory function; required full mech vent 24 hours, vent assist 30 days; treated with equine antitoxin | Urgent C-section at 36 weeks | Respiratory tract infection, drug withdrawal |
| Magri (2006)^25^ | Ham: type B | 28 years/26 weeks | Intubated, complicated by pneumonia | Spontaneous delivery at 40 weeks | None |
| Leclair (2013)^26^ | 2 patients: type E;  1 patient: type A | 3 patients: 1 in first trimester; others not specified | 1 patient: persistent toxemia for ≥10 days in first trimester. Other not specified. | 3 patients delivered healthy infants | None |
